# Supplementary material for: Exploring Interindividual Variability in Resilience to Stress: Social Support, Coping Styles, and Diurnal Cortisol in Older Adults
Source: Behav Sci (Basel). 2025 May 6;15(5):631. doi: 10.3390/bs15050631 (PMC12109149; doi:10.3390/bs15050631)
Supplement: Supplementary file 1 [file behavsci-15-00631-s001.zip › behavsci-3579940-supplementary.pdf]

Supplementary material

Table S1. Pearson's correlation coefficients between the cortisol index and the variables in our multiple regression models.

Variables	*M* (*SD*)/%	CAR	AUC	Peak-to-Bed
Control				
Body mass index	27.41 (5.85)	−0.029	0.023	−0.014
Medication use	43.7%	−0.14	−0.202 *	−0.185 *
Related to aging				
Age	76.26 (6.99)	0.047	0.174 *	0.090
Sex (female = 1)	88.1%	0.067	0.045	−0.011
Income		0.023	0.067	0.061
>$20,000	12.6%			
$21,000–$40,000	34.8%			
$41,000–$60,000	26.7%			
$61,000–$80,000	11.9%			
$81,000–$100,000	5.9%			
<$101,000	8.1%			
Years of education	13.73 (3.30)	0.065	0.008	0.011
Health-related QOL	39.11 (9.74)	0.215 *	0.177 *	0.212 *
Comorbidities	3.23 (2.17)	−0.066	−0.071	−0.115
Living situation (alone = 1)	60.7%	0.011	−0.050	−0.046
Subjective stress				
Major life event	1.49 (1.43)	−0.006	0.120	−0.001
Perceived stress	1.65 (0.62)	−0.106	−0.035	−0.137
Coping strategies				
Proactive coping	2.74 (0.33)	0.119	−0.063	−0.045
Strategic planning	2.76 (0.50)	0.095	−0.061	−0.009
Reflexive coping	2.85 (0.35)	−0.013	−0.067	−0.052
Preventive coping	2.97 (0.37)	0.091	0.047	0.066
Avoidance coping	2.83 (0.50)	−0.122	−0.140	−0.095
Social support				
Network availability	19.33 (9.25)	0.029	−0.031	0.038
Network satisfaction	5.07 (1.11)	0.068	0.018	0.041
Emotional support seeking	2.89 (0.50)	0.126	−0.019	0.082
Instrumental support seeking	2.81 (0.48)	−0.029	−0.056	−0.006

Note. CAR = cortisol awakening response; AUC = area under the curve with respect to the ground. * p-value < 0.05.
